# Quantified impacts of post-fire debris flows on the habitat, population, and recovery of the endangered black abalone

**DOI:** 10.1038/s41598-026-47783-1

**Published:** 2026-05-06

**Authors:** Wendy K. Bragg, Karah N. Cox-Ammann, Nathaniel C. Fletcher, Peter T. Raimondi

**Affiliations:** Department of Ecology and Evolutionary Biology, University of California Santa Cruz, 115 McAllister Way, Santa Cruz, CA 95060 USA

**Keywords:** Extreme climatic events, Land-sea connectivity, Post-fire debris flow, Impact assessment, Remote sensing, Intertidal, Black abalone, *Haliotis cracherodii*, Climate sciences, Ecology, Ecology, Environmental sciences, Ocean sciences

## Abstract

Research on emergent ecological effects of climate change has largely focused on incremental shifts. Meanwhile, extreme climatic events (ECEs), predicted to increase in frequency and severity, produce extraordinary stressors that may be instrumental in determining whether and to what extent species can adapt to incremental environmental shifts. The inherent unpredictability of ECEs temporally, spatially, and in intensity often precludes quantification of impacts, leaving important knowledge gaps to effective resource management and conservation. Here, we identify one trans-ecosystem driver of marine change via multiple terrestrial ECEs (fire + flood = debris flow). After early recognition of the potential for fire-induced impacts to endangered black abalone habitat, we applied predictive modeling to gauge site-specific risks which enabled us to prioritize sites for ephemeral pre-disturbance data collection. Following impacts from multiple post-fire debris flows, we applied a novel model that coupled in situ sampling with long-term monitoring data and aerial imagery to quantify impacts. We attribute a loss of 21% of rocky intertidal habitat and a median population loss of 59.6% of black abalone within the study area to these events. Results highlight the potential for ECEs to disrupt ecological processes in intertidal systems and offer a pathway to meaningful quantification of impacts in the wake of unpredictable and uncontrollable events.

**Supplementary Information:**

The online version contains supplementary material available at 10.1038/s41598-026-47783-1.

## Introduction

Globally, climate change is proceeding at an unprecedented rate^1–3^. While the ecological implications of comparably gradual trends have received considerable attention^4–6^, there is increasing recognition that punctuated environmental extremes can strongly influence the outcomes of a changing environment for species^7–10^. Extreme climatic events (ECEs) are rare, intense, and severely damaging climate-based events capable of causing dramatic ecological impacts^10–12^. The potential effects of ECEs on both terrestrial^9,13^ and marine^5,14–17^ ecosystems are gaining attention. However, studying these less-predictable ECEs presents distinct challenges^18^. Adding to the inherent complexities of ECE research, occasionally two or more extreme events co-occur simultaneously or sequentially as “compound ECEs.” Compound ECEs increase the potential for severe impacts due to their interactive effects^19,20^. The interactions between ECEs can create novel conditions, such as new hazards, including those spanning biomes^8,21^.

The coalescence of escalating and expanding numbers of stressors can challenge species persistence, especially for specialists, those already experiencing severe population declines, and species on the edges of shrinking ecological niches^22^. Predictions for increasing frequency and magnitude of ECEs^12,18,23^ highlight the importance that managers better understand and plan for such events to maximize successful resource management^10^. Yet, the unpredictability of ECEs in time, space, and extent leave many studies with insufficient data to quantify outcomes^24^. Short warning times, safety concerns, limited personnel and funds, and uncertainty surrounding predictions for impact locations, types, and extents can leave research teams with incomplete datasets. We suggest that careful consideration of the seemingly incohesive patchwork of disparate data types may reveal creative ways to unite them to assess impacts.

Here, we quantify impacts to marine ecosystems from two terrestrial compound ECEs that create a third ECE in the marine ecosystem (i.e., fire + flood = debris flow). Fires are chiefly terrestrial processes, however their impacts are not necessarily habitat-contained. Fires remove vegetation, alter soil properties, and can create water-repellent subsurface soil layers, thereby leaving landscapes prone to erosion^25–30^. Elevated risks for sedimentation can linger for months to years^25,31–35^. If heavy or prolonged rainfall follows, dangerous debris flows can result^36–42^. This relationship is likely to grow in import considering global climate model predictions of increasing size, intensity, and frequency of wildfires^6,43–47^; an increasing likelihood of extreme rainfall^6,7,48,49^; and more prevalent debris flows^29,50–53^. While initiation of post-fire debris flows depends on attributes within burned areas, debris flows themselves can rapidly transport up to 35 times the average downslope sediment to areas kilometers away from burn scars^30,35,54^. This pattern leaves fire-adjacent coastlines and their inhabitants prone to elevated sediment loads. Recently, remote sensing has been used in novel ways to examine wildfire-driven disturbances in marine environments^30,55–57^. To date, marine-focused studies of debris flows have focused on the pattern of sediment transport^30,58,59^ on sandy shores^30,59^ or subtidal areas^58^. This work revealed a pattern of initial offshore fluvial deposition followed by alongshore spreading^30^. Here, we hypothesized that coast-adjacent fires followed by heavy rain would compound to produce debris flow that could bury rocky intertidal shores and impart both immediate (via deposition) and subsequent (via littoral drift) mortalities of inhabitants.

Fires and droughts, punctuated by extreme storm events, are prevalent in Mediterranean environments and are expected to increase with climate change^52,60,61^. In central California, the Dolan Fire consumed 50,000 + ha (500 km^2^) of grassland, chaparral, and mixed evergreen forest immediately adjacent to the Big Sur coastline^62,63^ between August and December 2020. The area’s steep topography^54^ and the immediate proximity of the fire to the ocean further elevated concerns for post-fire debris flows in coastal ecosystems. The fire also aligned with the majority of the extant U.S. mainland population of black abalone^64^ (*Haliotis cracherodii*^65^, an endangered marine mollusk^66^, prompting efforts to proactively identify at-risk sites for pre-disturbance surveys (Supplemental Fig. 1). We collected baseline data from sites along the 39 km fire-adjacent coastline prior to 28 January 2021, the date when an atmospheric river dropped ~ 38 cm of rain in 2 days^67^, triggering numerous debris flows. Preliminary aerial imagery (Supplemental Fig. 2) suggested that debris flows inflicted extreme changes to rocky intertidal shores. One week later, tide levels and site conditions enabled access to impacted sites where we delineated three general zones: (1) the center, focused around basin outlets, where up to 3.5 m of sediment buried rocky intertidal habitat (Supplemental Fig. 3); (2) transition areas, extending tens to hundreds of meters up and downcoast, where all living materials (e.g., algae, invertebrates) had been completely scoured away, leaving bare rock “islands” protruding from sediment; and (3) adjacent areas beyond the footprint, where sediment had yet to affect habitat but where we determined there was an elevated risk of burial and scouring as littoral processes moved materials. We expected to document mortality in all three zones. Mortality in Zone 1 could be attributed to direct impacts, including smothering and injury from burial (Supplemental Fig. 4). That in adjacent Zones 2 and 3 could result either from secondary lethal effects of habitat loss but unrelated to complete burial (including starvation, desiccation, and predation, Supplemental Fig. 4) or transient burial which may go undetected in point estimates from aerial imagery.

While documenting post-disturbance conditions with uncrewed aircraft systems (UAS, i.e., drones) and field surveys, our team located and rescued a small subset of black abalone (primarily from areas described by #2 and 3 above). Over the next two years, we expanded monitoring efforts as sediment migrated and impacts manifested into distant and previously unaffected areas (Supplemental Fig. 5). Our findings emphasize the potential for compounded terrestrial ECEs to dramatically affect marine environments and the need to better understand the long-term implications of such events as climate change progresses. This study fills three critical gaps. First, our primary goal was to estimate losses attributable to this one event. To our knowledge, this study offers the first quantifiable estimate of interconnected ecological (black abalone) and structural (rocky intertidal habitat) marine impacts of post-fire debris flows on rocky shores, including mortality risk variability by degree of habitat degradation. Next, we explored how such unpredictable, yet episodic events may serve not only as drivers of local mortality but also may influence population distribution and have implications for species persistence and recovery. Finally, our study is a model for injury (mortality) assessment in the wake of ECEs.

## Materials and methods

### Study area and species

**Fig. 1:**
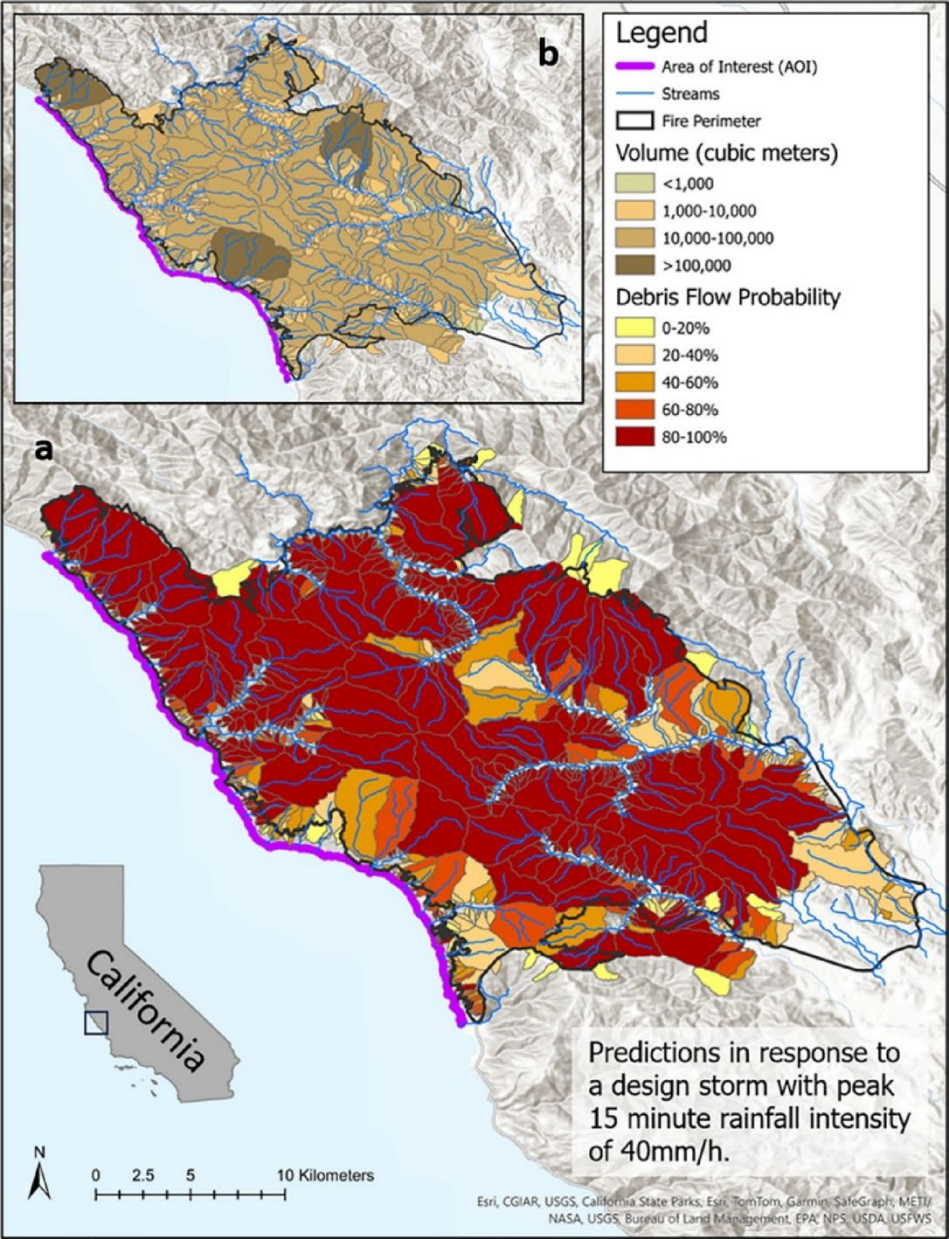
Overlaying USA Detailed Rivers and Streams layer^73^ and USGS Post-Wildfire Debris Flow Hazard Assessment Map^72^ to identify at-risk sites. **(a)** The main map displays the probability of debris flows originating in each basin based in response to a design storm with a peak 15-minute rainfall intensity of 40 mm/h. **(b)** The upper inset displays the predicted debris flow sediment volume in each basin.

The study area was along the Big Sur coast in central California, U.S.A., adjacent to the 2020 Dolan Fire (Fig. 1). This region is characterized by the rugged Santa Lucia Mountains that rise directly from the rocky shoreline of the Pacific Ocean to elevations of up to 1,500 m (Supplemental Fig. 1). Wildfires are common in the area, partially due to the steep slopes, dry climate, and mix of vegetative communities^35^. The Dolan Fire burned more than 50,000 ha (500km^2^, with a 39 km long western boundary that was consistently within 100–300 m of the ocean. The steep slopes that drop directly to the sea and the proximity of the fire to the coast left little intervening land to absorb sediment pulses.

Black abalone (Supplemental Fig. 1) are a marine mollusk endemic to the west coast of North America from Point Arena in northern California, U.S.A. to Bahia Tortugas and Isla Guadalupe, Mexico^68^. They are typically found in deep crevices and cracks or tight spaces formed by the underside of boulders in complex rocky intertidal habitat, from mean higher high water (MHHW) to −6 m relative to mean lower low water (MLLW)^69,70^. Black abalone were listed as endangered in 2009^66^ after populations were decimated by a combination of overharvesting and a fatal wasting disease called “withering syndrome”^70,71^. As of 2020 an estimated 70% of the known extant U.S. mainland population was thought to live along the Big Sur coast^64^.

### Data collection

Our primary goal was to quantify black abalone mortality due to post-fire debris flows. To achieve this, we compared the number of abalone present prior to disturbance (hereafter ‘pre’) with the number that remained after (hereafter ‘post’). This required information about both the representative abalone densities and the geomorphology of the coastline in each time period. Density data was assessed by sampling field plots in situ; geomorphology was assessed through remote sensing (Table 1). Disturbance is defined as the post-Dolan Fire debris flows that occurred on 28 January 2021. We assumed all abalone that were buried by sediment died, an assumption supported by post-disturbance surveys. Deposited sediment was likely to migrate^30^, so we incorporated coastline changes over a two-year period to assess disturbance effects. We also expected mortality in adjacent unburied areas, either due to secondary lethal effects or due to transient burial which went uncaptured in our imagery. An overview of methods and data types are displayed visually in Supplemental Table 1.

**Table 1:** Summary of data collected and sources for each data type.

Population Data:
(1) Number of black abalone in each sampled plot (field surveys)
Habitat Data:
(2) Geomorphology Class for coastline segments (remote sensing - coarse delineation)
(a) Pre-disturbance Period
(i) Baseline Rocky Shore (reexamined for fate of habitat in post-disturbance period)
(ii) Baseline Sandy Beach (masked from post-disturbance consideration)
(b) Post-disturbance Period
(i) Rocky Shore (Baseline Rocky Shore that persisted as rock in post period)
(ii) Sandy Beach (Baseline Rocky Shore covered by new sandy beach in post period)
(3) Coverage Category (remote sensing - fine examination of sampled plots in imagery)
(a) Rocky Shore all Rock (RS/R)
(b) Rocky Shore with Sand (RS/S)
(c) Sandy Beach with Rock (SB/R)
(d) Sandy Beach all Sand (SB/S)

#### Field survey design

Pre-disturbance field surveys began in October 2020 as a precautionary measure to obtain baseline data for use in the event of subsequent impacts. We identified 7 accessible at-risk sites (see *Sect. “*Selection of survey sites”*.*), where we established a total of 28 plots in rocky habitat (see *Sect. “*Plot design”*.*). Pre-disturbance field surveys were completed prior to 28 January 2021 to assess baseline black abalone density. For the following two years, most plots remained buried. We repeated sampling in May-November 2023 to capture post-disturbance conditions.

##### Selection of survey sites

We describe below our general methods for identifying candidate sites for sampling. However, due to the sensitive nature of endangered species management, specific location information cannot be shared or identified on maps.

**Fig. 2:**
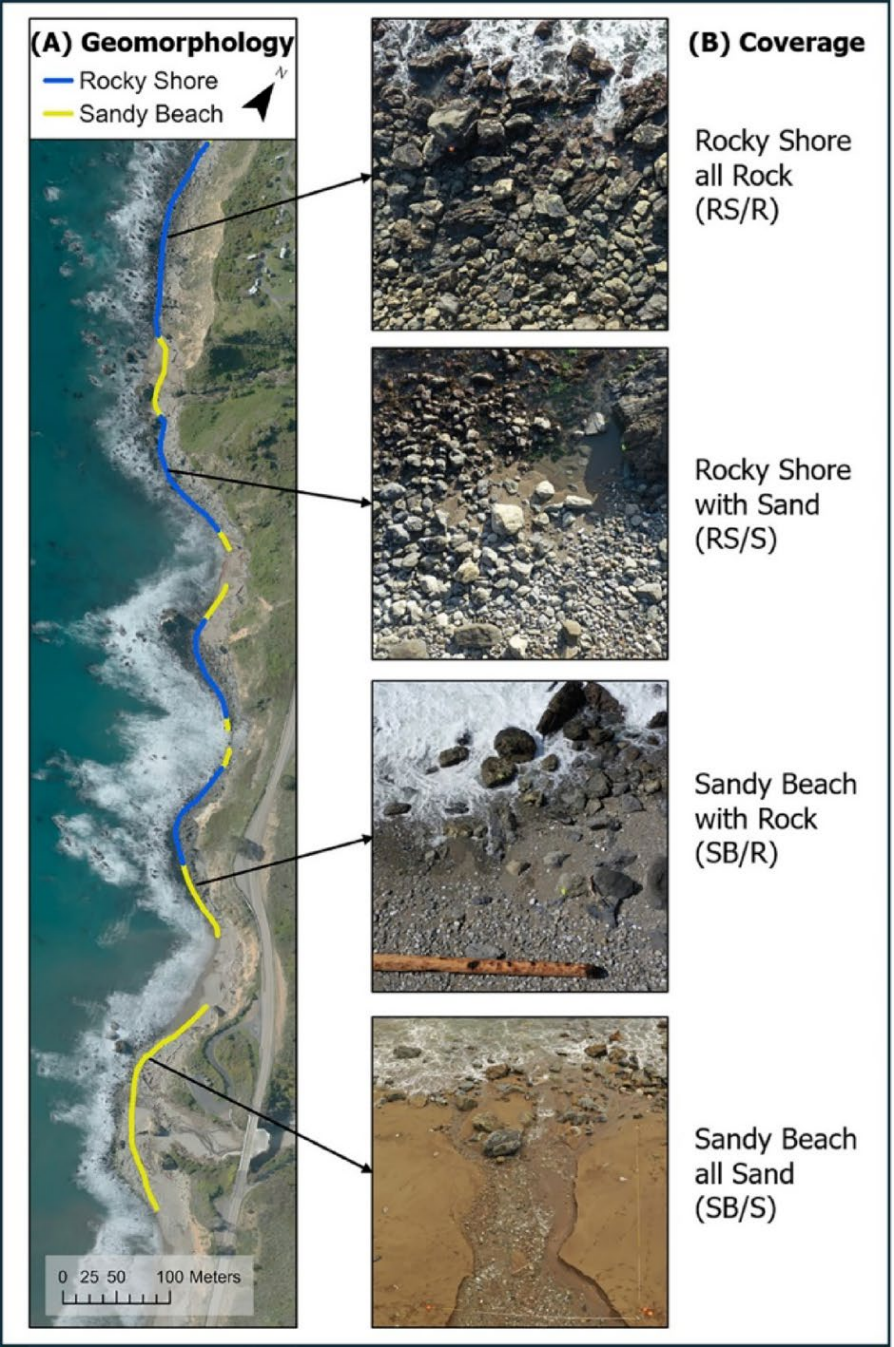
Examples of coarse delineation and fine examination of orthomosaics. **(a)** Coarse post-disturbance geomorphology delineation of a portion of the Area of Interest and **(b)** UAS images of representative plots illustrating four coverage categories (images are at approximately the same scale). Plots assigned to RS/R and RS/S are both found within shoreline coarsely classified as Rocky Shore. Plots assigned to SB/R and SB/S are subcategories within shoreline coarsely classified as Sandy Beach. The orthomosaic was constructed in AgiSoft Metashape version 2.0.3 (https://www.agisoftmetashape.com/) using USGS Remote Sensing Coastal Change (RSCC) project imagery^78^. The map was produced in Esri ArcGIS Pro version 2.8.8. (https://www.esri.com/en-us/arcgis/products/arcgis-pro/overview).

We combined the USGS Post-Wildfire Debris Flow Hazard Assessment Map (hereafter ‘Hazard Map’) of the Dolan Fire^72^ with the USA Detailed Rivers and Streams layer^73^ to: (1) define an Area of Interest (AOI) that encompassed the full coastline at increased risk for debris flows (Fig. 1 and 2) identify specific river basin outlets within the AOI where we anticipated elevated sedimentation could occur (hereafter ‘at-risk sites’). The Hazard Map divides the burn scar into polygons (basins) within which basin shape, burn severity, soil properties, and rainfall characteristics are used to estimate the probability (Fig. 1a) and volume (Fig. 1b) of debris flow occurrences in response to design storms^74^. Potential sediment loads from individual basins (which would compound as they merged into river channels) ranged from 10,000 to 100,000 + m^3^ (Fig. 1b). To gauge risk level of coastal sites, we appraised the hazard level of polygons that fed into a given waterway and identified the coastal outlet of that waterway. This produced a list of at-risk sites that were ranked by compounded hazard level of upstream polygons. From this list, we targeted sites that were both rocky shoreline and were accessible by land. Thus, sampling sites were necessarily non-randomly allocated along the AOI.

##### Plot design

Coastlines are a mix of bedrock, boulders, and varying sizes of smaller particles. At the coarsest level, black abalone ‘habitat’ consists of rocky intertidal coastline, dominated by bedrock and boulders (jointly ‘rock’). ‘Nonhabitat’ consists of beaches dominated by small particles such as cobble, sand, and gravel (jointly ‘sand’). Researchers avoided beaches and established all plots in rocky habitat in at-risk sites. Importantly, rocky shore and sandy beach are readily identifiable in aerial imagery.

At each selected site, we established a series of rectangular plots in a geometric distribution centered around the river mouth (Supplemental Fig. 6) to capture what we predicted to be a gradation of impacts, with greatest effects expected closest to the outlet and decreasing with distance. Established plots encompassed the full searchable intertidal range for black abalone (MHHW to water’s edge during negative low tide; a tidal elevation range of 1.5 m to 2 m) and were mapped and marked with marine epoxy to allow relocation for subsequent sampling.

From the river mouth, the first plot in a series was established at the nearest 10 m (measured parallel to shoreline) of rocky habitat deemed appropriate for black abalone. Subsequent plots were located 10 and 100 m from this first plot, for a total of 3 plots in the series (ideally 0 m, 10 m, 100 m). Interval distances were not altered except to avoid locating a plot on sand or because geology precluded access. The series was mirrored upcoast and downcoast of the outlet, totaling up to 6 plots at each site. Plot locations were established without prior knowledge of abalone presence, allowing for unbiased sampling of abalone density.

The alongshore length of plots was uniformly 10 m, but width (from MHHW to water’s edge) varied from 3 m to 20 m (mean = 12.4 m, SD = 5.7 m) based on physical characteristics of the shoreline. Because of variability in plot area, all density data are reported as “abalone per *linear* meter” of rocky shore, which was calculated by dividing the total count of abalone in each plot by 10 m. This method is standard for black abalone density calculations by the Multi-Agency Rocky Intertidal Network (MARINe)^75,76^ and was also necessary for abundance calculations (Eq. 1) using linear meters of coastline geomorphology (Sect. 2.2.2.2).

##### Plot sampling data

We surveyed plots in accordance with methods developed by MARINe, a long-term rocky intertidal monitoring consortium^77^. A team of researchers with extensive experience monitoring black abalone thoroughly searched each plot to count abalone. Counts were used to characterize the density of black abalone (per linear meter) that typified the conditions of rocky shores during the pre and post periods.

#### Remote sensing design

Remote sensing entailed two scales of imagery analysis: coarse and fine. Coarse delineation revealed geomorphology classes that reflected the physical properties of the shoreline. Fine examination of each field-sampled plot enabled characterization of the range of sediment coverage.

##### Aerial imagery selection and processing

The United States Geological Survey (USGS) regularly flies the California coast to monitor coastal processes using an oblique plane-mounted camera system. We used this imagery to build orthomosaics for change detection analysis within the AOI. We divided USGS aerial data from 2020 to 2023 into pre and post intervals with a separation date of 28 January 2021. From the available data^78^, we selected four flight dates (two pre and two post) based on temporal proximity to debris flows, appropriate tide level (≤ −0.03 m or ≤ −0.10 ft MLLW), and sufficient lighting to differentiate intertidal geomorphology (Supplemental Table 2). Images were collected with a Nikon D810 camera in RAW format. We used AgiSoft Metashape version 2.0.3 (https://www.agisoftmetashape.com/) to construct orthomosaics with 15 cm resolution. Co-alignment followed a ‘4D-SfM’ technique^79^ and the USGS imagery processing workflow^80^. The AOI aligned with the coast adjacent to the Dolan Fire.

##### Coarse delineation of coastline geomorphology

Our goals for coarse delineation of coastal geomorphology were twofold: (1) to estimate pre-disturbance extent (meters) of rocky shore (habitat) that existed within the AOI, and (2) to identify how much of that habitat persisted versus became buried after debris flows. We used these measurements to project field-sampled abalone densities to the complete AOI.

We censused the coastline within the AOI to coarsely delineate the shore into geomorphology classes, based on physical properties. Areas of rocky shore could potentially support black abalone occupation (habitat), while sandy beaches were deemed incapable of supporting black abalone (nonhabitat). Pre imagery was delineated into either ‘Baseline Rocky Shore’ or ‘Baseline Sandy Beach’. Because Baseline Sandy Beach was not deemed habitable and, therefore, would not contribute to baseline populations, it was masked from post-disturbance consideration. Areas classified as Baseline Rocky Shore were reexamined in post imagery and delineated into either areas that persisted as rocky habitat (hereafter ‘Rocky Shore’) or areas that began as habitat but were subsequently covered by debris flows (hereafter ‘Sandy Beach’) (Fig. 2a). Delineation was accomplished in three steps using Esri ArcGIS Pro version 2.8.8. (https://www.esri.com/en-us/arcgis/products/arcgis-pro/overview): establishing a template, analyzing orthomosaics, and merging data.

First, we digitized the AOI to create a template polyline feature fitted to the shoreline at roughly MHHW. This was overlaid onto each orthomosaic to standardize the area examined. Recognizing that coastline conditions are never static, we utilized two flight dates for each time period (Supplemental Table 2). For each orthomosaic, two researchers independently traced the template polyline, delineating segments into rocky shore or sandy beach, with a threshold of ten (10) contiguous meters to switch classification categories. This produced a set of polyline features that represented the coastline geomorphology for each flight. Finally, each researcher’s polylines were merged for the two pre flights and for the two post flights to create three composite feature classes: Baseline Rocky Shore, Rocky Shore, and Sandy Beach. This merging was tailored for pre and post periods.

In the pre-period, given the inherent dynamics of coastlines, especially where rocky shore and sandy beach meet, we determined that locations deemed sandy beach in *both* pre flight dates would be assigned to Baseline Sandy Beach (nonhabitat) and masked from post-disturbance consideration (since they would not have contributed to baseline population numbers), while locations that were rocky shore in *either* pre flight date would be assigned to Baseline Rocky Shore.

For post-period, we defined the loss interval as the two years following debris flows, beyond which time we judged that persistent coastal processes introduced an unacceptable level of uncertainty to our ability to identify sedimentation sources. We used two flight dates as point estimates of disturbance conditions (Supplemental Table 2). The first date reflected immediate debris flow coverage attributed to direct riverine processes. The second informed where sediment subsequently moved due to longshore transport. To encompass both immediate and subsequent impacts, we merged polylines from these two point estimates such that locations classified as sandy beach in *either* post flight date were assigned to Sandy Beach, while locations classified as rocky shore in *both* post flights were assigned to Rocky Shore.

This three-step process produced two estimates each of Baseline Rocky Shore, Rocky Shore, and Sandy Beach meters (one for each researcher) for use in our models.

##### Fine examination of plot sediment coverage

The goal of fine examination was to identify whether coarsely delineated geomorphology classes were internally homogenous. To more closely discern sediment that might be present in areas delineated as rocky shore, we overlaid polygons of the field-sampled plots onto each post-period orthomosaic. Researchers independently examined this subset of imagery for each date at a zoomed-in (fine) scale to discern the state of sediment coverage (‘coverage’) for each plot at that time point, without regard to the underlying coarsely assigned geomorphology (Fig. 2a). Categories included: ‘all Rock’ (no meaningful coverage), ‘mix of Rock and Sand’, or ‘all Sand’ (complete sediment coverage). Then, the two coverage categories from these time points were compared and the most extensive (highest coverage) of the two was retained and assigned to all associated data (pre and post) as a label for aggregation. While coverage classification was only revealed by the post-period state of plots, applying the label to both pre and post periods allowed us to aggregate data based on eventual sedimentation effects for direct comparison. These labels do not imply that pre period plots had sediment coverage.

From this, we discerned that impacts were not binary. Rather, ‘mix of Rock and Sand’ existed in both post-disturbance Rocky Shore and Sandy Beach, therefore generating four coverage labels (Fig. 2b; Table 2): Rocky Shore all Rock (hereafter ‘RS/R’), Rocky Shore with Sand (hereafter ‘RS/S’), Sandy Beach with Rock (hereafter ‘SB/R’), and Sandy Beach all Sand (‘SB/S’). While RS/S indicates areas that were coarsely classified as Rocky Shore but revealed to contain some sand, SB/R indicates predominantly Sandy Beach areas containing some exposed rock. As described below, we calculated and applied the proportions of these categories when making calculations.

**Table 2:** Geomorphology classes and coverage categories. Orthomosaics were first examined coarsely to delineate geomorphology classes (Baseline Sandy Beach was masked from post-period consideration). Field plots had been established and sampled in a subset of the coastline. Polygons of these plots were overlaid on post orthomosaics and reexamined at a fine scale (without regard to underlying geomorphology class). Researchers discerned varying degrees of within-plot sediment coverage: no meaningful coverage (‘all Rock’), ‘mix of Rock and Sand’, and complete coverage (‘all Sand’). Because ‘mix of Rock and Sand’ existed in both Rocky Shore and Sandy Beach, four (4) coverage categories were revealed.

Geomorphology Class (Coarse delineation of AOI)		Coverage Category (Fine examination of plots to assess sediment coverage; applied to aggregate data)		
Pre	Post	all Rock	Mix of Rock and Sand	all Sand
Baseline Rocky Shore	(Persistent) Rocky Shore	(Persistent) Rocky Shore all Rock (RS/R)	(Persistent) Rocky Shore with Sand (RS/S)	-
	(New) Sandy Beach	-	(New) Sandy Beach with Rock (SB/R)	(New) Sandy Beach all Sand (SB/S)
Baseline Sandy Beach	Masked from post-disturbance consideration			

### Analytical approach

Our analyses addressed two questions. First, we estimated total black abalone and habitat losses. Second, we examined how mortality risk varied between coverage categories.

We applied abalone densities (generated by sampling plots) to geomorphology classes (generated by censusing aerial imagery of the entire AOI), aggregating data by coverage categories (extent of sedimentation, Table 2). Only plots sampled in both pre and post intervals, making them pairable, were included in analyses (*N* = 28). Because plots were not balanced among our four coverage categories, we calculated the proportions of each and applied these to our calculations.

All data and analytics that support our results are available online^81^.

#### Black abalone and habitat losses

The primary goal of this study was to estimate black abalone losses. Because these estimates were derived from habitat changes, we also generated an estimate of habitat loss.

##### Model overview and equation

We combined data from two different sampling approaches in our model to estimate losses. One approach (field sampling) was applied to determine mean density estimates of black abalone in rocky habitat (biology-based). The other approach (remote sensing) provided data on the physical condition of the habitat, without any information about abalone (geomorphology-based; Fig. 2a). To enable projection of biological data from the field-sampled subset of the AOI across the complete AOI, we united these data types by closely examining imagery of field-sampled plots to assess the degree of sedimentation (coverage) that occurred in this subset of habitat (Fig. 2b). Coverage contributed to our model in two important ways. First, it gave us an estimate of the proportions of our AOI that had been differently affected by debris flows (Table 3), allowing us to adjust geomorphology meters accordingly for calculations. Second, it allowed us to aggregate plots and associated data into similarly affected groups to assess how abalone densities and habitat changed in each of those types of areas. This aggregation also served to minimize noise originating from any inherent differences between plots.

**Table 3:**
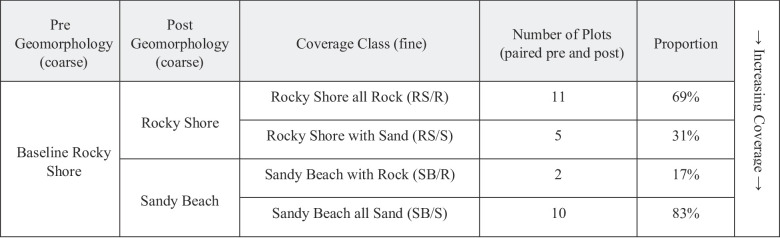
Coverage categories, number of plots, and calculated proportions. Number of plots determined to be of each coverage category were used to generate four proportions that were applied to geomorphology lengths for loss calculations. Because RS/R and RS/S were both nested within coarsely classified Rocky Shore, while SB/R and SB/S were nested within coarsely classified Sandy Beach, we calculated the coverage category proportions such that: % Rocky Shore all Rock + % Rocky Shore with Sand = 100% Rocky Shore. % Sandy Beach with Rock + % Sandy Beach all Sand = 100% Sandy Beach.

The underlying equation (Eq. 1) that was used to calculate both the pre and post abundances is:

1 $$\:\:\:\:\:\:\:\:{\sum}_{i}{\left(Gc\right)}_{i}{D}_{i}\:= Total\: Abundance\: at\: a \:time\: interval \:summed \:over \:coverage\: categories \:i.$$

Where:

(Gc)_*i*_ = Coverage-specific geomorphology meters.

D_*i*_ = Coverage-specific black abalone linear density.

*               i* = Coverage category (*Rocky Shore all Rock; Rocky Shore with Sand; Sandy Beach with Rock; Sandy Beach all Sand)*.

To estimate losses, we subtracted paired data within each coverage category (post minus pre) to generate four coverage-specific losses, which we summed to produce an estimate of Total losses.

Our goal was not only to produce a distribution of estimates of mean losses (ȳ’s) but to generate an associated confidence level that our estimated mean includes the true mean (*μ*). Due to the uncontrolled nature of this study (dependent on unpredictable natural forces), coverage classes were not balanced (Table 3). Thus, applying a simplified version of Eq. (1) (i.e., using only the sampled means) would have involved multiplying terms with different sample sizes, different variances, and without known covariances. This approach would have yielded an estimate of mean loss but would have complicated an estimation of a confidence interval. To remedy this, we bootstrapped (with replacement) the measured variables (geomorphology meters and abalone density) for use in Eq. (1), yielding a distribution of 2500 means that represented the array of possible means (ȳ) that could have come from the population of interest.

##### Key Variables

We based our model on methods developed by Raimondi et al^75^. which we modified to incorporate geomorphology changes and coverage categories. Our model allowed us to apply biological data to the full AOI in a way that was representative of the observed levels of habitat change by combining three variables: abalone density (D, field sampling), geomorphology meters (G, remote sensing), and proportion of coverage category (c, united field and remote sampling).

**Black abalone linear Density (D).** Plot data were aggregated by coverage to generate four sets of coverage-specific abalone densities (D) for each time period (pre and post). These eight datasets were bootstrapped, with replacement, for use in Eq. (1). The post densities of all plots classified as Sandy Beach all Sand were recorded as zero, even if areas were not searchable due to ongoing burial.

**Geomorphology meters (G).** Two estimates (one for each researcher) of the meters of Baseline Rocky Shore, Rocky Shore, and Sandy Beach were bootstrapped, with replacement, for use in Eq. (1).

**Proportion of coverage category (c).** We calculated the proportion of coverage category (c), such that:

*% Rocky Shore all Rock + % Rocky Shore with Sand = 100% Rocky Shore*.

and

*% Sandy Beach with Rock + % Sandy Beach all Sand = 100% Sandy Beach*.

Coverage category proportions (Table 3) were not bootstrapped themselves because each was a point estimate. Rather, they were used to adjust bootstrapped geomorphology meters (G) as described below.

##### Applying the equation

Equation (1) was applied to each of the 2500 iterations to calculate Total and Percent abalone losses. To estimate Total losses, we first multiplied bootstrapped geomorphology meters (G) by the appropriate proportion of coverage category (c) to generate 2500 iterations of geomorphology meters for each of the four coverage categories (Gc). Next, we calculated the product of coverage-specific geomorphology meters (Gc) and the corresponding coverage-specific density (D) to generate an estimated abalone abundance for each coverage category. This yielded four pre and four post coverage-specific abundances for each of 2500 iterations. Among the 2500 iterations, some chance combinations paired a smaller pre abundance with a larger post abundance, which would have generated negative “losses”. Since negative losses are, by definition, impossible, we set post abundance equal to pre abundance for these pairings, essentially yielding losses that would all be ≥ 0. We termed these adjusted post-abundances “zero-corrected post abundances”. Then we calculated zero-corrected loss within each of the four coverage categories by subtracting the corresponding estimates of abundance (pre minus zero-corrected post), yielding four coverage-specific zero-corrected losses for each of 2500 iterations. Finally, we added the four coverage-specific zero-corrected losses to generate 2500 estimates of Total loss.

We used the above calculated Total losses to estimate Percent losses by summing the four pre-period coverage-specific abundances to generate a total pre-period abundance for each of 2500 iterations. We divided Total loss by the associated total pre-period abundance, yielding 2500 estimates of Percent loss.

In all, the above steps generated 2500 estimates of Total loss (number of abalone) and 2500 estimates of Percent loss.

#### Sedimentation attributes associated with mortality risk

For each coverage category, we divided the mean zero-corrected loss by the respective mean black abalone pre abundance to generate four coverage-specific estimates of the likelihood for mortality under varying degrees of sedimentation (termed ‘mortality risk’).

## Results

### Black abalone and habitat losses

**Fig. 3:**
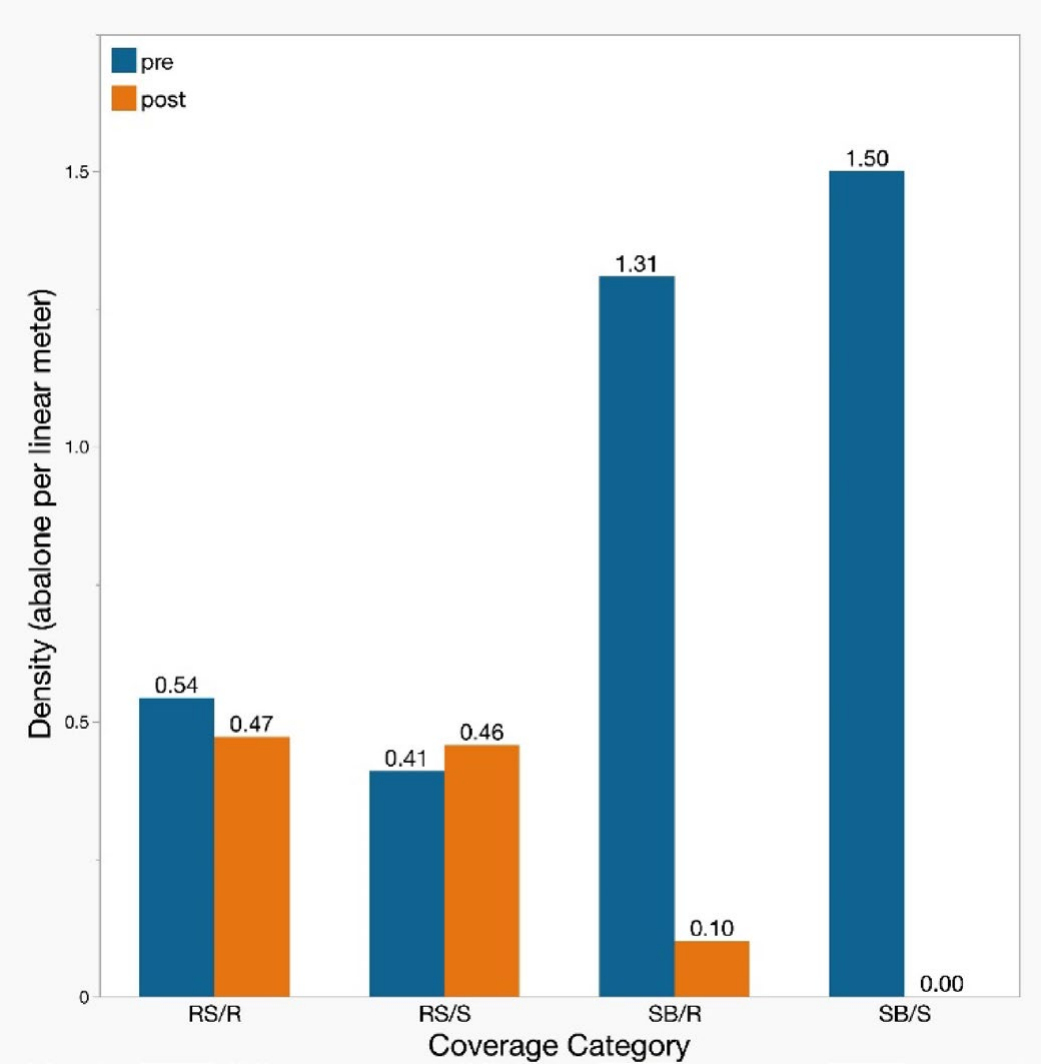
Coverage-specific mean abalone linear density for plots established in baseline rocky habitat. Plot black abalone linear density was measured during pre- and post-disturbance periods. Note: all plots began as Baseline Rocky Shore and were later transformed to varying degrees by debris flows (see Fig. 2). Each plot was assigned a coverage category label that reflected the most extensive sediment coverage recorded for the plot over the course of the study (see Table 2). Plots were aggregated into the four coverage categories and abalone density data for pre- and post-disturbance periods were compared within each coverage category as a measure of change. From left to right are coverage categories: Rocky Shore all Rock (RS/R), Rocky Shore with Sand (RS/S), Sandy Beach with Rock (SB/R), Sandy Beach all Sand (SB/S).

Abalone losses were calculated using geomorphology meters (Table 4), proportion of coverage category (Table 3), and black abalone linear density (Fig. 3).

**Table 4:** Geomorphology meters. Two researchers coarsely examined the template polyline feature on each of four orthomosaics to delineate geomorphology classes. Data from two flight dates were merged to generate mean geomorphology composite meters for: Baseline Rocky Shore, Rocky Shore, and Sandy Beach. Due to very slight overlapping of segments during delineation, columns of Rocky Shore and Sandy Beach did not sum exactly to Baseline Rocky Shore.

Researcher	Date	Sample Period	Baseline Rocky Shore (m)	Rocky Shore (Rock Retained) (m)	Sandy Beach (Rock Lost) (m)	Percent Habitat Loss Sandy Beach ÷ Baseline Rocky Shore
1	19 Mar 2020	Pre	33,627	-	-	-
	10 Jan 2021					
	26 Mar 2021	Post	-	26,644	6,975	21
	17 Mar 2023					
2	19 Mar 2020	Pre	33,683	-	-	-
	10 Jan 2021					
	26 Mar 2021	Post	-	26,537	7,142	21
	17 Mar 2023					

**Fig. 4:**
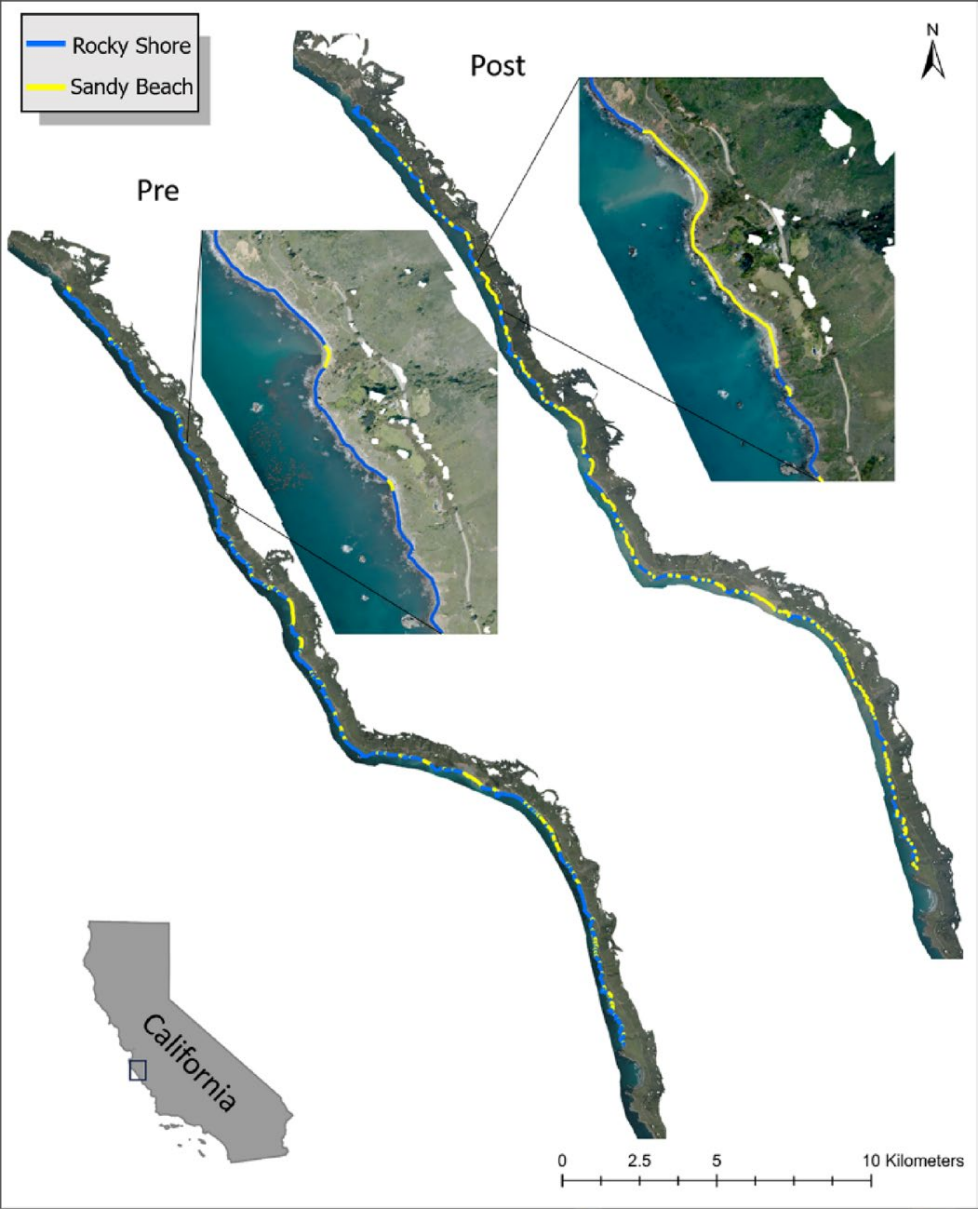
Pre and post coastline classification showing the change in geomorphology due to post-fire debris flows. Insets show a zoomed in view of one impacted area to illustrate the change in sand coverage. Orthomaics were constructed in AgiSoft Metashape version 2.0.3 (https://www.agisoftmetashape.com/) using USGS Remote Sensing Coastal Change (RSCC) project imagery^78^. Orthomosaic coordinates are in North American Datum of 1983-National Adjustment of 2011 (NAD83(2011)) and projected to Universal Transverse Mercator (UTM) Zone 10 N coordinates in metric units. The map was produced in Esri ArcGIS Pro version 2.8.8. (https://www.esri.com/en-us/arcgis/products/arcgis-pro/overview).

We used geomorphological changes (Fig. 4) to estimate population losses via habitat loss. From Table 4, we estimate a mean loss of 7,059 m (21%) of Baseline Rocky Shore.

**Fig. 5:**
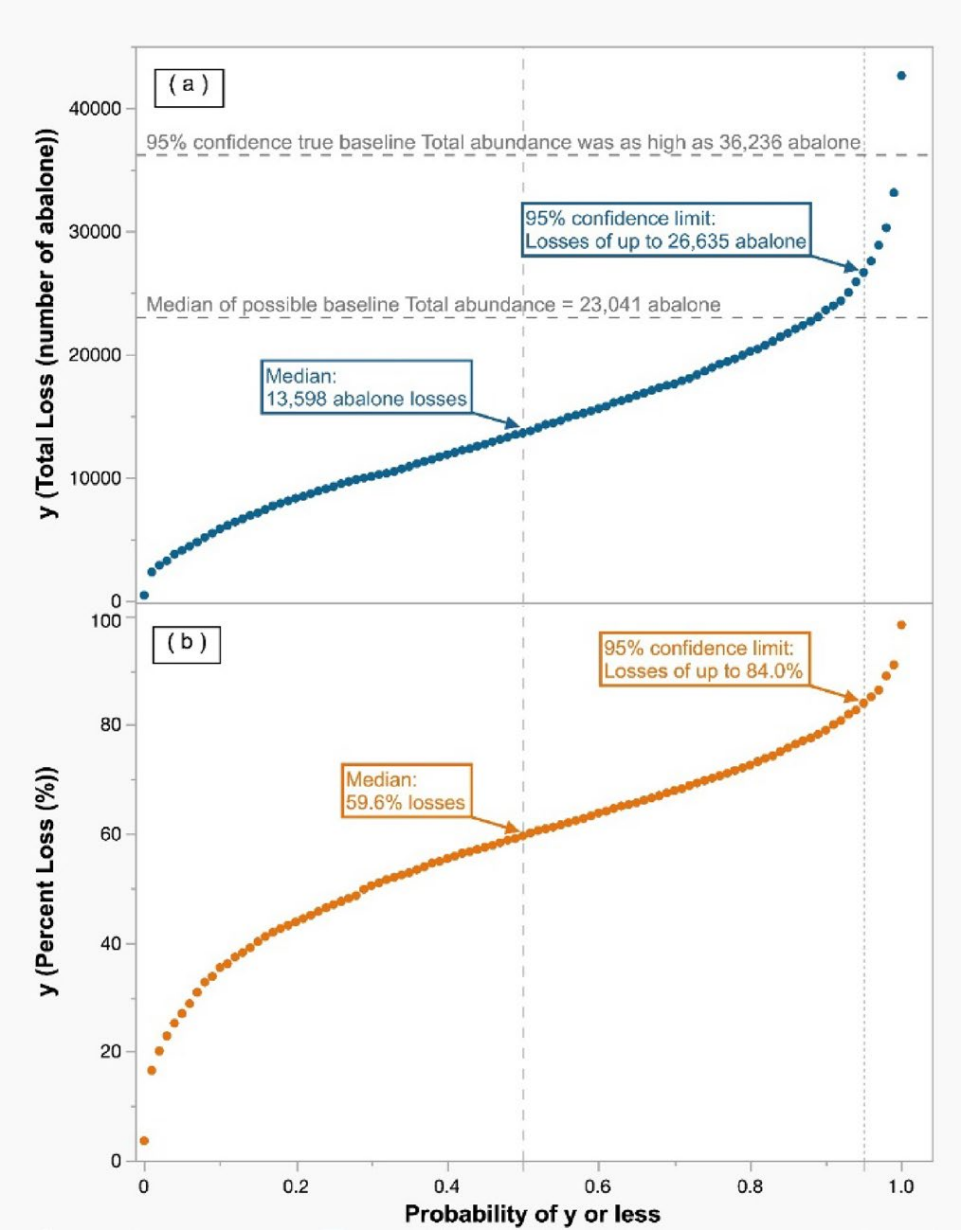
Probability (x-axis) of corresponding black abalone losses (y-axis). The figure shows the cumulative distributions of bootstrapped coverage-specific estimates of black abalone Total Loss **(a)** and Percent Loss **(b)**. Confidence limits for the true mean (*μ*) can be directly read off the x-axis. The most likely estimates of true loss (*μ*) are indicated by the textbox medians: 13,598 abalone or 59.6% of the baseline population. The upper 95% confidence limit textboxes indicate that true losses (*μ*) may have been as high as 26,635 abalone or 84.0% of the baseline population. Horizontal dashed lines on panel (a) were added to reflect baseline population reference levels: median (23,041 abalone) and 95% confidence limit (26,635 abalone).

Bootstrapping the measured variables (geomorphology meters and abalone density) for use in Eq. (1) generated 2500 estimates of Total loss and 2500 estimates of Percent loss. We present these loss estimates in Fig. 5 as cumulative distributions to show the range of possible values for true loss (y-axis) and the corresponding probability of that level of loss or less on the x-axis. This representation allows direct interpretation of confidence limits from the x-axis. Two places are of particular interest. First, the median of estimated loss (ȳ), representing the best estimate of true loss (*μ*), was calculated to be 13,598 abalone or 59.6% of the pre-disturbance population. Additionally, the upper 95% confidence limit indicates that the true mean (*μ*) may have been as high as 84% (26,635 abalone) of the baseline population.

### Sedimentation attributes associated with mortality risk

Table 5 presents four mean coverage-specific metrics: baseline abundance, zero-corrected loss, percent of total loss (zero-corrected loss divided by the sum of all losses), and mortality risk (zero-corrected loss divided by baseline abundance).

**Table 5:** Coverage-specific means. Data were aggregated into the four coverage categories and bootstrapped to generate mean coverage-specific metrics including baseline abundance, zero-corrected loss, percent of total loss (calculated by dividing zero-corrected loss by 14,338, which is the sum of all four zero-corrected losses), and mortality risk (calculated by dividing zero-corrected loss by baseline abundance).

Pre Geomorphology (coarse)	Post Geomorphology (coarse)	Coverage category (fine)	Mean Baseline Abundance	Mean Zero-Corrected Loss	Percent of Total Loss	Mortality Risk (%)
Baseline Rocky Shore	Rocky Shore	Rocky Shore all Rock	9,929	2,963	21%	30%
		Rocky Shore with Sand	3,408	1,096	8%	32%
	Sandy Beach	Sandy Beach with Rock	1,541	1,450	10%	94%
		Sandy Beach all Sand	8,829	8,829	62%	100%

## Discussion

Our work suggests substantial and specific ecological susceptibility to the forces of ECEs and offers an example of capitalizing on available data to produce scientifically sound estimates of impacts. Following the 2020/2021 post-fire debris flows, our team was left with diverse datasets that were each individually insufficient to assess impacts to black abalone and its habitat. Developing a novel approach to unite these disparate data, we estimated that as much as 84% of the baseline population (Fig. 5) and 21% of the baseline habitat adjacent to the Dolan Fire (Table 4) were lost in this one event. These quantified estimates of considerable losses from a newly identified threat both highlight the potential of extreme events to disrupt ecological processes and help managers make better-informed choices when strategizing the recovery of the species.

From aerial imagery, we estimated a mean habitat loss of 21% within the AOI (Table 4). We believe this represents a conservative estimate of habitat loss (as well as the derived population loss) since sediment may have impinged and retreated from additional habitat between flight dates, causing losses that escaped capture by imagery.

Our methodological approach, uniting biological data with these measured physical changes, indicates that the most likely (median) black abalone losses were 59.6% of the baseline population (Fig. 5). Perhaps more concerning for managers, results indicate with 95% confidence that true losses may have been as great as 84.0% of the population (Fig. 5).

Predictably, the highest numeric losses and mortality risk (loss per baseline abundance) (Table 5) were associated with completely buried areas (SB/S) where there was 100% mortality. However, these areas accounted for only 62% of total mortality, suggesting other contributing factors. Indeed, previous studies compiled by Airoldi document non-burial negative effects of sedimentation^82^. Further examination revealed two themes. First, mortality risk varied most dramatically between geomorphology classes, such that mortality risk of the two coverage categories nested within Rocky Shore (30% in RS/R and 32% in RS/S) were markedly different from those nested within Sandy Beach (94% in SB/R and 100% in SB/S). Secondly, 29% of Total losses were attributed to areas that would have been deemed unimpacted via remote sensing analysis alone (coarsely delineated as Rocky Shore). Viewed together, these observations suggest that, while burial operates as the key driver of mortality, additional forces likely underlie declines, a nuance that may have been missed if only one data type were considered. Possibilities include: (1) areas in Rocky Shore were buried and uncovered without being captured by imagery, and/or (2) elevated stressors from habitat degradation (e.g., changes to community, food-availability, scouring, etc.) led to mortality and/or emigration of animals. The lack of suitable intervening imagery made assessment of the former impossible. However, based on frequent onsite visits to conduct UAS flights (Supplemental Fig. 5), we believe it is unlikely that sediment inundation substantial enough to affect coarse delineation (geomorphology class) went uncaptured on a large scale. Thus, we assume the most likely causes of declines in Rocky Shore were habitat degradation and associated mortality (Supplemental Fig. 4) and/or emigration. Loss due to emigration could include movement to subtidal habitat (black abalone range as deep as 6 m) or to areas adjacent to impact zones. While our data cannot directly address this point, we believe it to have contributed minimally to population changes. Abalone are slow moving and black abalone, in particular, are relatively sedentary (long term monitoring shows individuals in the same location over many years)^64^ and initial burial occurred too rapidly to have permitted emigration. Moreover, emigration in advance of secondary inundation likely was limited by the extensive sediment coverage, which created islands of habitat (rock) isolated by a ‘sea’ of sediment (Supplemental Fig. 4- upper left). If animals did move subtidally, this likely provided only limited refuge as researchers observed evidence of subtidal impacts (e.g., dead subtidal invertebrates, including red abalone (*Haliotis rufescens*) and gumboot chitons (*Cryptochiton stelleri*), and subtidal algae drift found washed ashore at locations impacted by debris flows) throughout the study period. Therefore, we suspect that observed declines more likely represent mortality than emigration. Field observations of impacts to the low intertidal provided some support for these assertions (Supplemental Fig. 7).

The effects we document were observed within an area considered to be the stronghold of mainland black abalone populations, from which scientists had hoped the species would expand and recover. Such substantial mortality (median 59.6%, Fig. 5) and habitat loss (mean 21%, Table 4) over two years, caused by one intersection of compound ECEs, suggest broader implications when placed in context of the longevity of primary population declines, potential secondary population limitations, losses relative to the entire Big Sur coast, and recovery of black abalone.

The more time that passes since the 2020/2021 fire + flood = debris flows, the more difficult it becomes to attribute ongoing changes to this one-time event. However, we suspect that primary population declines (those directly related to the forces of debris flows) may persist by means of: (1) longshore transport of already-deposited materials onto new areas of rocky habitat; (2) continued churning of elevated sediment loads, affecting even unburied areas through intensified scouring and similar associated stressors; and (3) additional terrigenous sediment inputs from the burn scar, which remains at elevated risk for erosion. Our study directly demonstrates the first two processes within the study timeframe and suggests a continuation, though at reduced severity. The third was evidenced by additional slides onto rocky intertidal habitat adjacent to the burn scar in 2024. Together, these factors further support the assertion that mortality estimates are conservative.

This study does not address the long-term consequences of population declines. However, black abalone reproductive strategy (broadcast spawning), requires dense aggregations of adults. Additionally, abalone have a relatively short pelagic larval duration, so recruitment is largely local^83,84^. Many impacted areas may reach a density threshold (< 1 abalone/m^2^)^85^ at which fertilization and subsequent recruitment cannot occur, severely limiting or preventing local recovery. Previous studies demonstrate that sedimentation can affect ecosystem shifts for invertebrates not only through reduced fertilization but also through reduced settlement and juvenile survival and through increased lethal mutations^82,86–91^. Thus, the effects of population declines will likely be further exacerbated by reduced future recruitment. Even as primary mortality effects of sedimentation dissipate, secondary effects (those resulting from population declines) may reach well into the future.

In 2020, an estimated 70% of the known extant U.S. mainland population of black abalone were thought to live along the Big Sur coast^64^. The losses we report here occurred within a 39 km subsection of that larger 175 km Big Sur coast. To determine the relative weight of these localized losses, we used the U.S. National Oceanic and Atmospheric Administration’s Environmental Sensitivity Index (ESI), Central California Shoreline Types layer^92^ to estimate the extent of rocky shoreline along the entire Big Sur coast. We then used the overall mean pre-disturbance density (abalone/m shoreline) from the AOI to estimate total black abalone abundance in the Big Sur region. We compared our calculated median losses (13,958 abalone) to the estimated Big Sur population size (109,007 to 140,604 abalone, see available dataset for calculations^81^) to estimate relative losses. Our calculations indicate that this one-time event resulted in a 10–12% loss of the entire Big Sur population.

Historical data suggest that black abalone have likely faced destructive sedimentation events throughout their history^30,93^. However, natural events (e.g., landslides and debris flows) are now joined by escalating anthropogenic effects (e.g., road construction, development, dredging, beach nourishment, and accelerated coastal erosion linked to climate change) to increase the variation, frequency, and intensity of sedimentation events faced by black abalone. Simultaneously, the species is confronted by anthropogenically linked disease events, poaching, contaminant spills, warming sea surface temperatures, ocean acidification, sea level rise, marine heatwaves, and coastal construction^70^. As with many species under increasing stress from a rapidly changing environment, the effects of ECEs may play a crucial role in determining winners and losers. For black abalone, the interplay between fire, extreme storms, and sediment appears poised to be increasingly influential in light of global climate predictions.

The insights gained from this study are directly applicable to black abalone management and point to the need to better understand both marine sedimentation hazards and the characteristics that identify landscapes as vulnerable to repeated impacts. Importantly, our results empower risk management, planning, and recovery implementation by managers. Key lessons include the need to identify low risk areas where restoration can be prioritized; to diversify recovery strategies aimed at bolstering broad regions of populations, thereby dampening the potential impacts of unpredictable pronounced losses following environmental disasters; and to expand long-term monitoring to enable efficient, effective, and data-supported injury assessment and/or response operations to future disasters. Such efforts offer to strengthen the species generally in the face of known and as-yet unknown threats to persistence.

Conducting injury assessment of the unplanned and uncontrollable compound ECEs (fire + flood = debris flows) presented unique challenges. We overcame the various challenges associated with each phase through tailored approaches. Initially, we leveraged long-term monitoring, experienced personnel, knowledge of potential fire-induced effects, and the lag time between fire ignition and rain event to identify at-risk sites and conduct anticipatory baseline surveys. Data collection (baseline, monitoring, and final) was further aided by the opportune coalescence of beneficial circumstances, including: funding that was flexible and substantial enough to support anticipatory surveys; a pre-existing multi-institutional network of experienced personnel who readily supported all phases of data collection; and the capacity to dedicate one person as a full-time project manager. Lacking certainty for which metrics would prove meaningful, our baseline dataset was necessarily diverse in an attempt to gather as much information as possible. After impacts, sedimentation became the clear metric of import. With limited time, personnel, and funding, we narrowed our focus to tracking sedimentation and associated effects to black abalone for post-disturbance sampling. Finally, to quantify losses, we assessed the available data and drew information from two very different data types: biological (field sampling) and geomorphological (remote sensing). To unite these diverse metrics, we conducted fine-scale imagery examination of field-sampled plots. The approach we then developed allowed us to generate robust quantitative estimates of injury with accompanying confidence limits under challenging, and data-limited conditions.

Our work serves as a model for accomplishing quantitative, scientifically sound injury assessment in the wake of ECEs. Recognizing that each circumstance will be distinctive to the affected ecosystem, impacting event(s), personnel, and other specifics, keys to success will likely be founded in identifying and leveraging the unique opportunities presented at each stage along the process. While all stages can present unique challenges, initial data collection in the aftermath of impacts will likely be among the most active phases of the process. In its wake, as personnel regroup, creative utilization of available data may be necessary to enable injury assessment. To increase the likelihood of informative outcomes, we encourage expanding long-term monitoring and strengthening collaborative relationships, both of which may prove irreplaceable in times of need.

## Conclusions

To our knowledge, this study offers the first quantifiable estimate of interconnected ecological (black abalone) and structural (rocky intertidal habitat) marine consequences of post-fire debris flows on rocky intertidal shores. This study quantifies substantial black abalone and habitat losses, explores ecosystem responses that may contribute to and exacerbate those losses, and suggests management implications. We focused on black abalone due to its primarily sedentary life history (precluding escape of impacts), its large size relative to other intertidal organisms (simplifying population counts), its endangered status, and the abundant long-term monitoring data that informed population estimates and species range. However, the implications of this study are germane to other species, other sediment events, and other ECEs.

Black abalone served as a proxy for the broader intertidal community. The observed impacts naturally affected the entire intertidal ecosystem and onsite observations suggested offshore and subtidal impacts as well. During reconnaissance, we noted the complete removal of biota (algae and invertebrates) from impacted intertidal areas (Supplemental Fig. 7), as well as substantial damage to offshore algae, and signs of subtidal invertebrate mortality. Limited personnel and funds along with elevated safety concerns, restricted our focus solely to intertidal impacts.

This study explored post-fire debris flows, but unprecedented rates of sedimentation have also been linked to agriculture^94^, mining^95^, dam removal^96,97^, deforestation^98^, development^99^, and dredging^100^. There are growing concerns for the potential of these actions to affect nearshore and offshore communities^101,102^. The substantial mortality we document stresses the need to better understand elevated sediment pulses, regardless of the source, to inform management considerations for at-risk recipient nearshore areas.

As global climate change continues to create novel challenges for ecosystems, understanding the complex implications of ECEs to ecosystem health and species persistence is increasingly important for developing effective management strategies in a changing world^103^. Prior to this study, subject experts had not expected such concerning levels of impacts to marine ecosystems from nearby fires. This highlights the need for unconventional considerations of developing and changing environmental conditions to identify and understand other as-yet-unknown risks to ecosystem health. In addition to predictable trends, scientists need to be prepared to examine sudden impacts. A key aid in this effort is long-term monitoring programs that provide broadscale baseline data as well as knowledgeable and skilled personnel able to rapidly shift to unpredictable developments such as ECEs. Long-term monitoring will likely grow in importance for identifying potential effects and measuring developments of both known and yet-to-be-discovered environmental threats and for assessing natural recovery compared to recovery following any human intervention actions. Equally as important is the ability to develop creative ways to use available data to produce sound measures of changes. We encourage others to undertake similar efforts to assess injuries in the wake of natural and anthropogenic disasters and to share the strategies they employ.

## Supplementary Information

Below is the link to the electronic supplementary material.

Supplementary Material 1

## Data Availability

The data and analytical approach that support the findings of this study are publicly available in Zenodo^81^.
