# Supplementary material for: Quantified impacts of post-fire debris flows on the habitat, population, and recovery of the endangered black abalone
Source: Sci Rep. 2026 May 6;16:21747. doi: 10.1038/s41598-026-47783-1 (PMC13358090; doi:10.1038/s41598-026-47783-1)
Supplement: Supplementary file 1 — Supplementary Material 1 [file 41598_2026_47783_MOESM1_ESM.docx]

**Supplemental Information For:**

### **Quantified Impacts of Post-fire Debris Flows on the Habitat, Population, and Recovery of the Endangered Black Abalone**

**Authors:**

Wendy K. Bragg^a*^, Karah N. Cox-Ammann^a^, Nathaniel C. Fletcher^a^, and Peter T. Raimondi^a^

^a^ Department of Ecology and Evolutionary Biology, University of California Santa Cruz, 115 McAllister Way, Santa Cruz, CA, 96050, USA

*Corresponding author:

Wendy Bragg

Department of Ecology and Evolutionary Biology

University of California Santa Cruz

115 McAllister Way

Santa Cruz, CA, 96050, USA

wbragg@ucsc.edu

+1-605-354-0269

**Supplemental Figures**


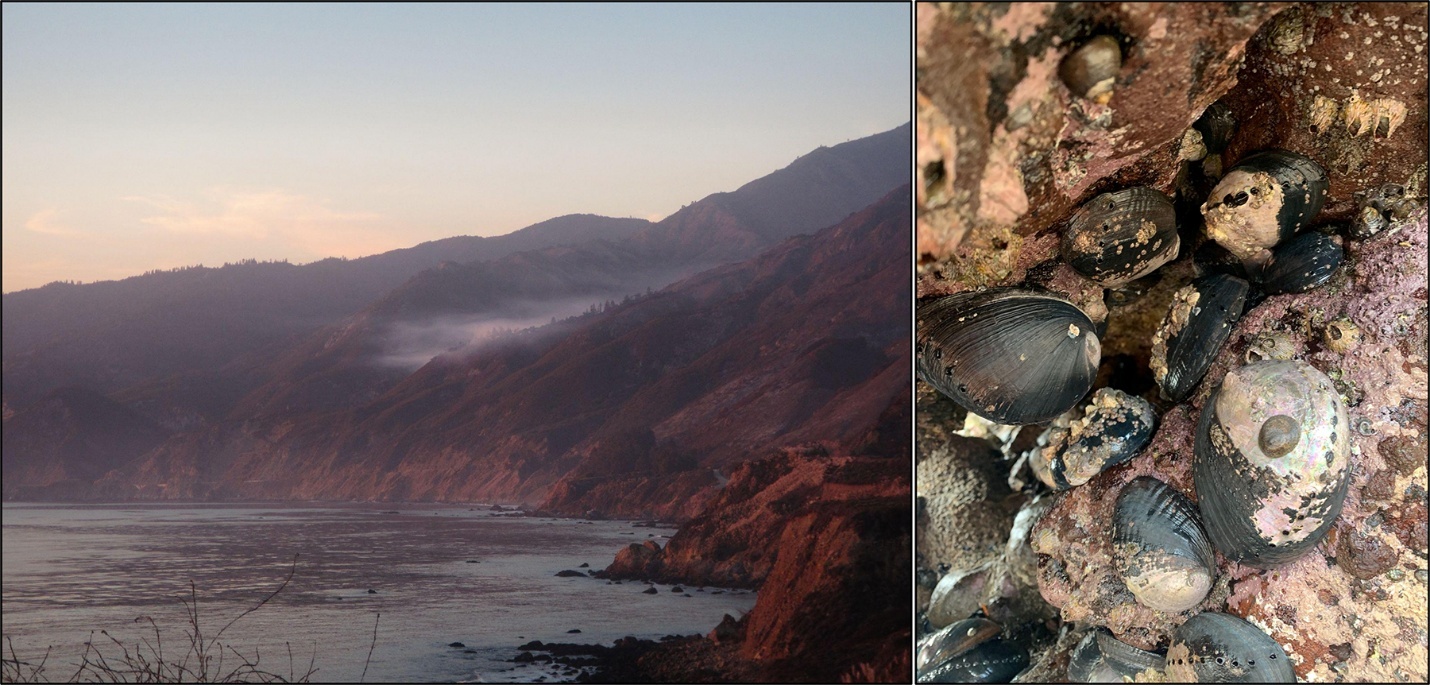


**Supplemental Figure 1. Big Sur and black abalone.** The Dolan Fire burned the steep San Lucia mountains in Big Sur, California from August to December 2020 (left, photo in October 2020, image credit: Wendy Bragg). Black abalone in characteristic crevice habitat (right, image credit: Nathaniel Fletcher).


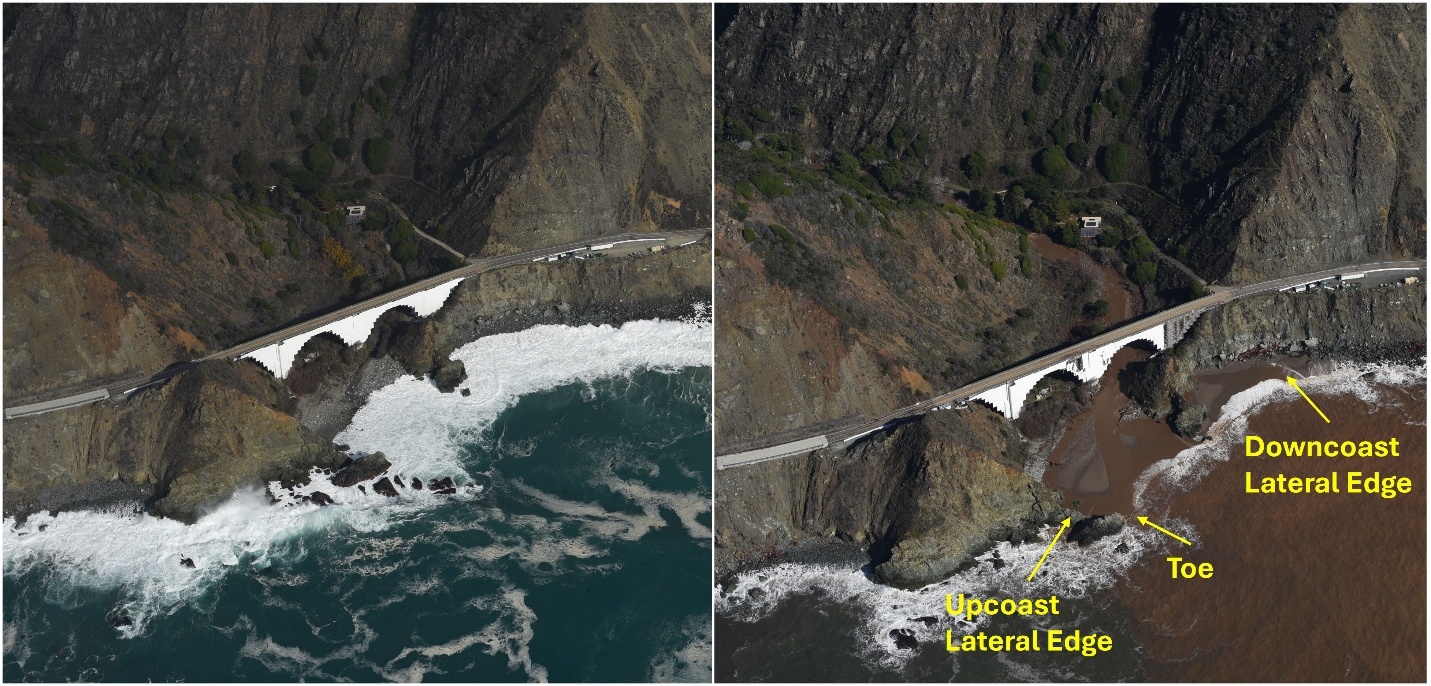


**Supplemental Figure 2. Aerial imagery at one location before and after debris flows, with annotations of debris flow morphology.** USGS aerial imagery^1^ on 10 January 2021 (left) and 29 January 2021 (right) show changes caused by post-fire debris flows that occurred on 28 January 2021.


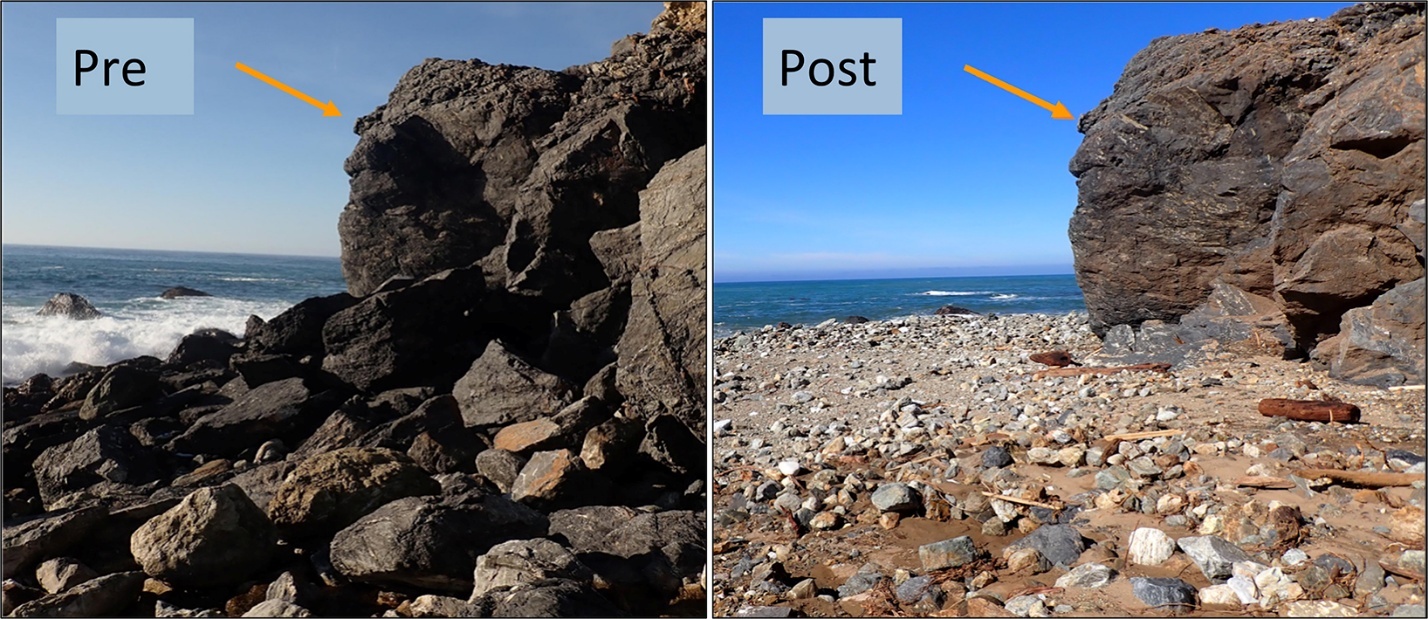


**Supplemental Figure 3. One study site before and after debris flows.** The photographer is standing at the river mouth, looking upcoast, on 14 January 2021 (left) and 7 February 2021 (right). Here, an estimated 3.5 meters of sediment from post-fire debris flows buried the rocky intertidal habitat on 28 January 2021. Image credit: Wendy Bragg.

**
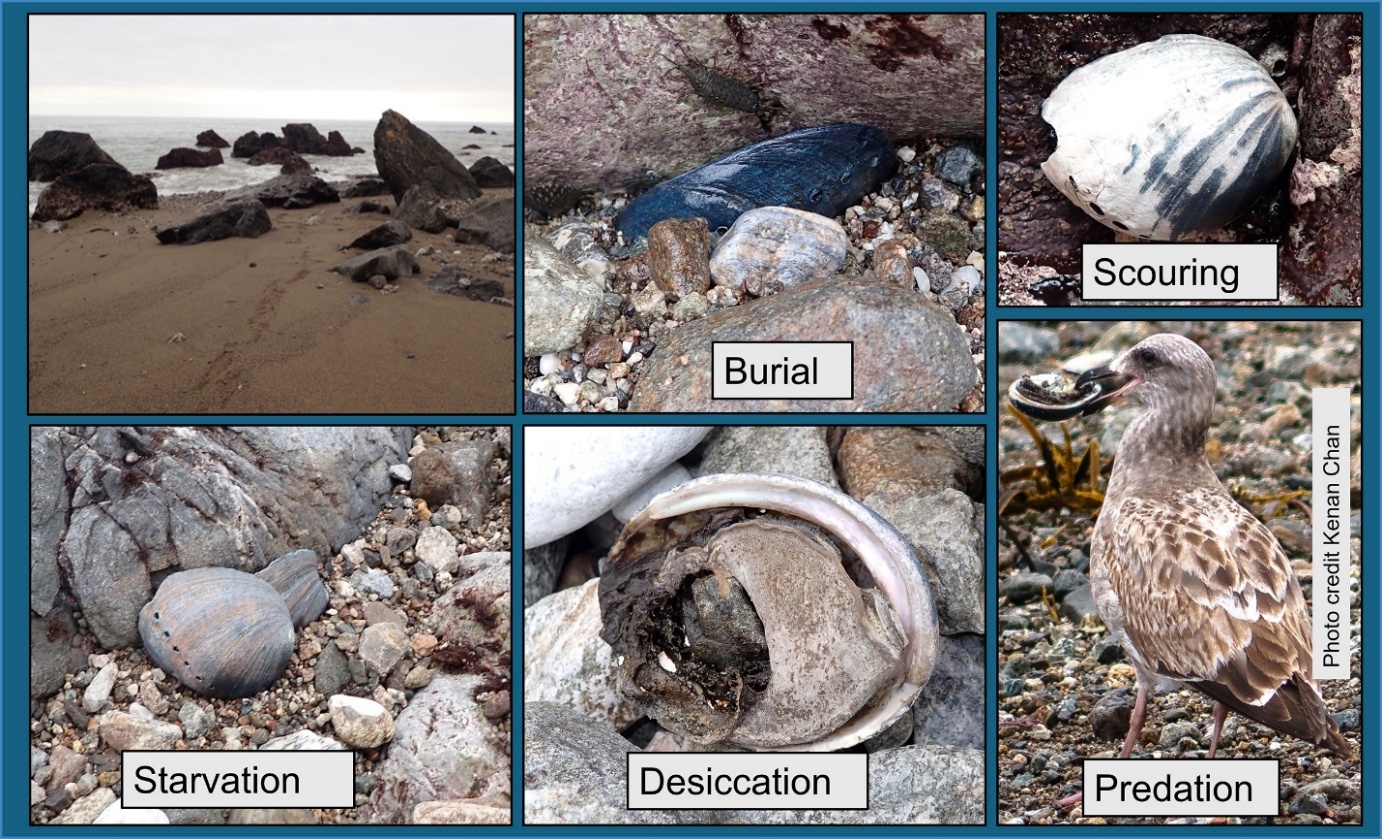
**

**Supplemental Figure 4. Photo documentation of observed post-fire debris flow habitat and stressors affecting black abalone.** Upper left photo shows example of inundated habitat. Direct stressors include partial to complete burial and scouring. Indirect stressors that result from being stranded far from the new high-water mark include starvation, desiccation, and predation. Image credits: Wendy Bragg and Kenan Chan (“Predation” photo).


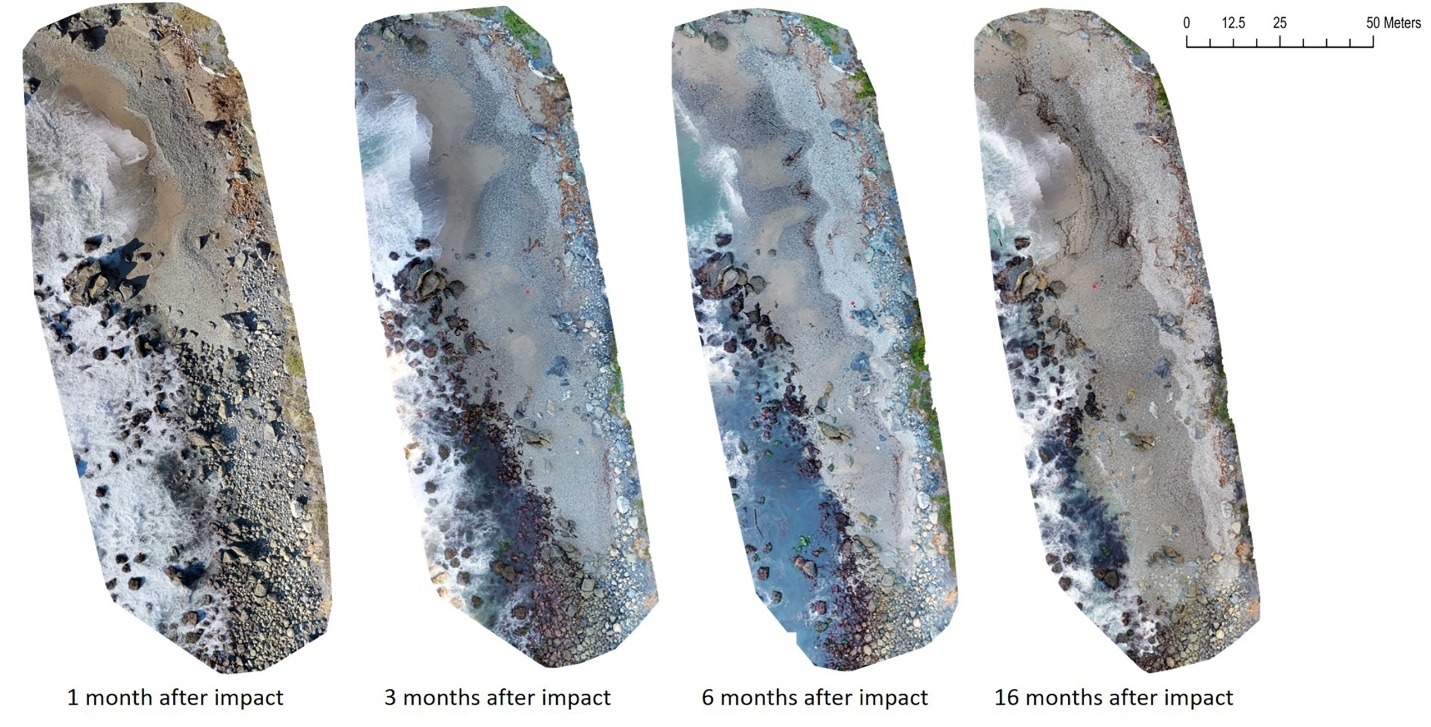


**Supplemental Figure 5. Time series of orthomosaics at one study site, indicating the temporal expansion of sediment coverage.** A DJI Mavic 2 Pro quadcopter drone equipped with a 20MP Hasselblad L1D-20c camera was used to track sediment load movement over a two-year period at three high-impact sites. Orthomosaics were produced in PIX4Dmapper photogrammetry software.


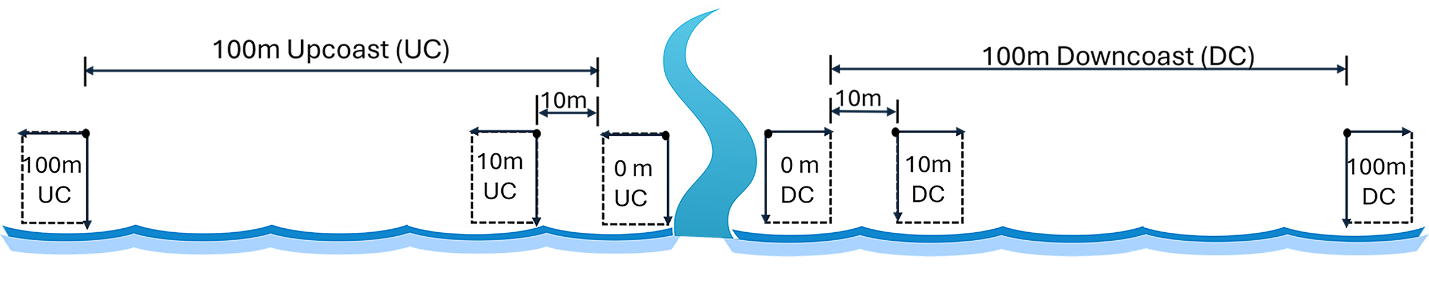


**Supplemental Figure 6. Diagram of plot series layout, centered around an at-risk river basin outlet.** Plots sampled at 0, 10 and 100 meters upcoast (UC) and downcoast (DC) of basin outlet. Length is measured alongshore; width is measured from mean higher high water (MHHW) to water line. (Design adapted from protocols detailed in MARINe Handbook^2^).


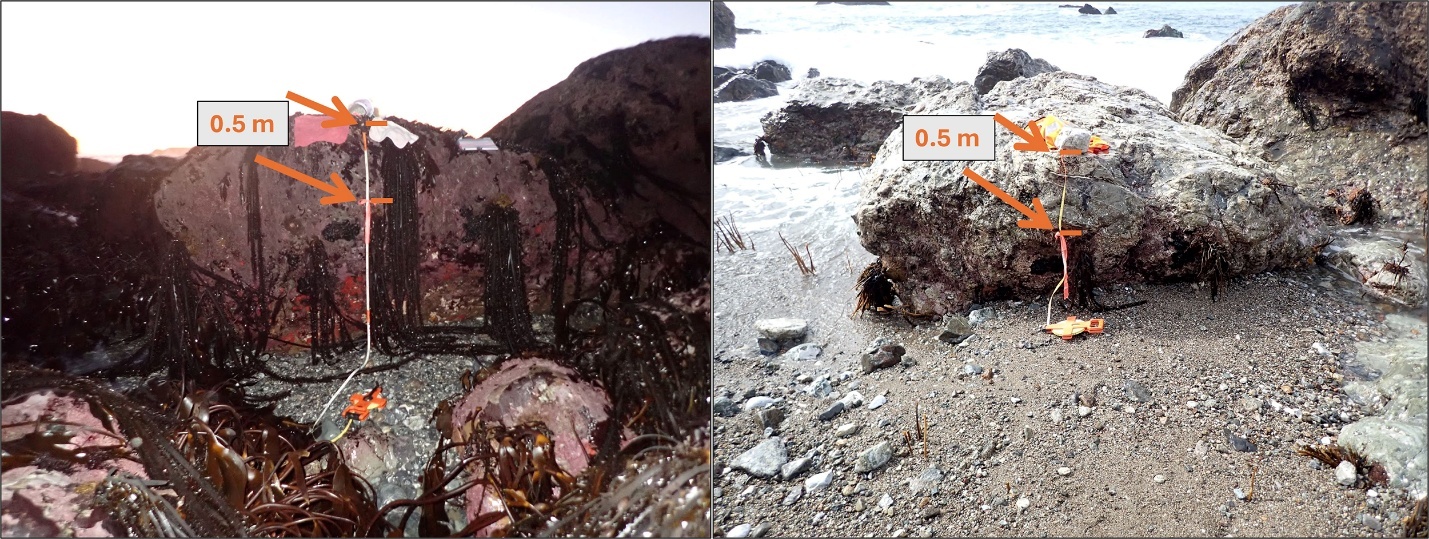


**Supplemental Figure 7. Removal of biota far from the impact zone center.** This marker rock is ~100 m upcoast of the river mouth. Prior to the debris flow, the rock and surrounding area supported substantial algae and other biota (left, 15 December 2020). The impacts of the debris flow resulted in near complete removal of biota even at this distance from the center of the impact zone (right, 23 February 2021). The top arrow shows the location of the installed reference point and the bottom arrow indicates where flagging marks 0.5 m below the reference point, highlighting the increase in sediment post-impact. Image credit: Wendy Bragg.

**Supplemental Tables**

**Supplemental Table 1.** Overview of study methods and data types utilized for change detection before (pre) and after (post) disturbance. Cell height reflects relative sample sizes of data categories.

**
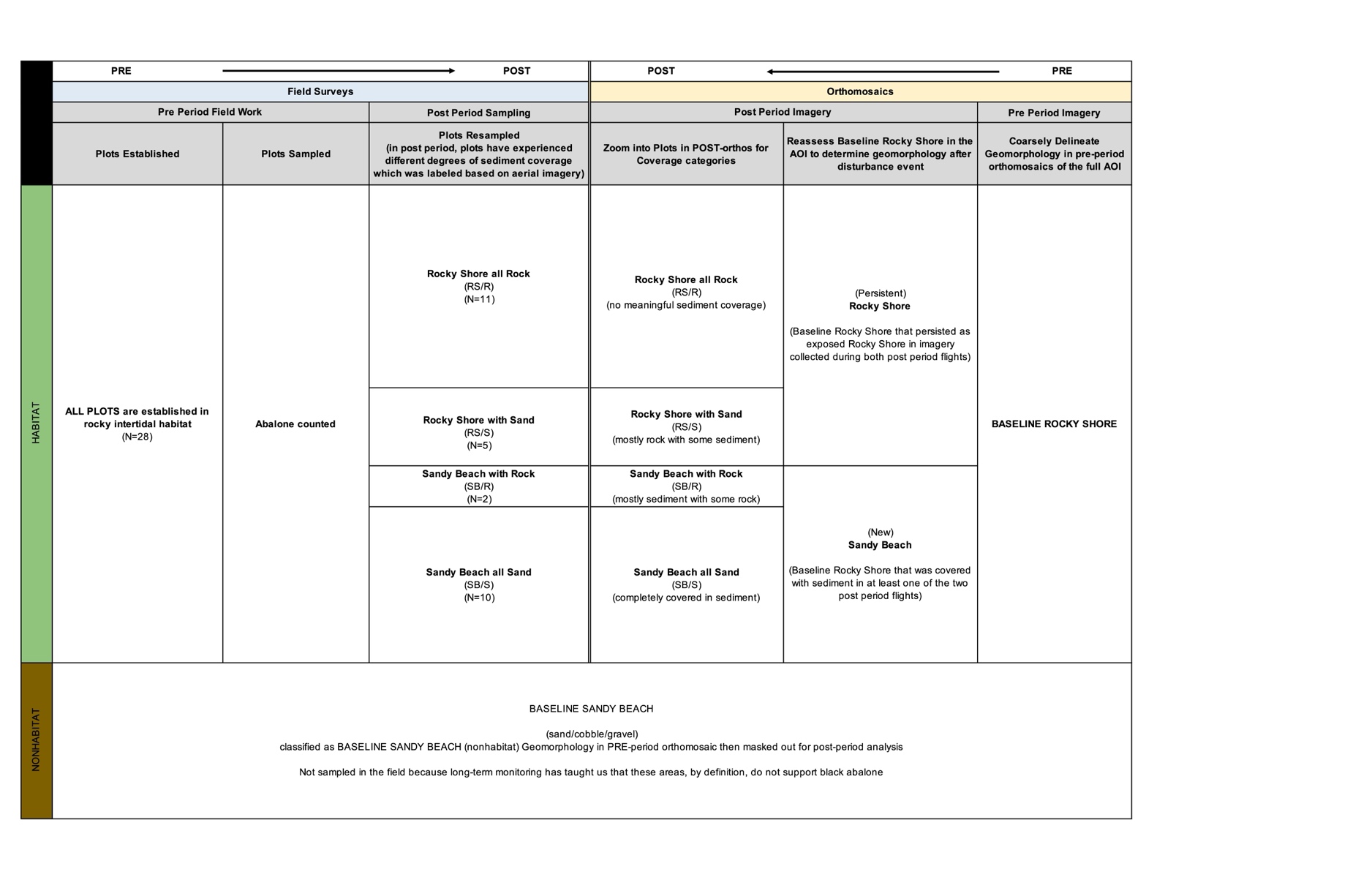
**

**Supplemental Table 2. Aerial imagery metadata.** Four USGS flights were chosen to create orthomosaics of the coastline adjacent to the Dolan Fire scar based on three characteristics: temporal proximity to the 28 January 2021 debris flows, appropriate Mean Lower Low Water (MLLW) tide level during the flight time, and sufficient daylight to differentiate the intertidal geomorphology.

| Flight Date | Time Period  (relative to Debris Flows) | Tide Level (m) MLLW | Time of Flight |
| --- | --- | --- | --- |
| 19 Mar 2020 | Pre 1 | -0.09 m | 1500 hr |
| 10 Jan 2021 | Pre 2 | -0.05 m | 1300 hr |
| 28 Jan 2021 | DEBRIS FLOWS | | |
| 26 Mar 2021 | Post 1 | -0.13 m | 1615 hr |
| 17 Mar 2023 | Post 2 | -0.03 m | 1245 hr |

**Supplemental References**

1. Ritchie, A. C., Triezenberg, P., Warrick, J., Hatcher, G., & Buscombe, D. D. PCMSC PlaneCam – Field data from periodic and event-response surveys of the U.S. West Coast, in Remote Sensing Coastal Change Simple Data Distribution Service: U.S. Geological Survey data service, accessed November 14, 2023, at <https://doi.org/10.5066/P9M3NYWI>. (2023)
2. Engle, J. M. *et al.* *Unified Monitoring Protocols for the Multi-Agency Rocky Intertidal Network*. <https://doi.org/doi:10.6085/AA/marine_ltm.15.3> (2022).
